# Exploring Consumer Perceptions and Changing Consumption Patterns for Smoked Paprika: Implications for Traditional Food Products in Spain

**DOI:** 10.3390/foods12142808

**Published:** 2023-07-24

**Authors:** Olda Lami, Celia Sama-Berrocal, Alberto Martín, Francisco J. Mesías, Rocío Velázquez

**Affiliations:** Escuela de Ingenierías Agrarias, Universidad de Extremadura, 06007 Badajoz, Spain; oldalami@unex.es (O.L.); celiasamaberrocal@unex.es (C.S.-B.); amartin@unex.es (A.M.); fjmesias@unex.es (F.J.M.)

**Keywords:** traditional food products, spices, paprika consumption, consumer perceptions, *Pimentón de La Vera* PDO paprika

## Abstract

Changes in the level of income of the population and a reduction in time availability are shifting food consumption from traditional to more convenient food products. The production of traditional food has a significantly positive impact on the territory, as it contributes to preserving cultural traditions and identity and supports rural development and resilience, thus becoming essential for the survival of rural areas. Within traditional food products, spices are among the most usual ingredients in traditional cuisine, extensively used to add flavour and colour to dishes. However, spices in general and paprika in particular—one of the most typical spices in the Spanish cuisine and the subject of this paper—have been rarely the subject of scientific studies. The purpose of this study is to analyse consumers’ perceptions towards paprika and determine the level of its consumption. For this purpose, a survey was conducted on a representative sample of Spanish consumers. The findings indicated that the average consumption of paprika was 154.5 gr/person per year. Although the general consumers’ perceptions toward this spice were generally positive, they proved to be less positive amongst young people and inhabitants of large cities, whose consumption of the spice was also lower. Additionally, the *Pimentón de La Vera* PDO paprika was the most popular version of paprika, being mentioned by all the participants. The findings from this study confirm the change in food consumption patterns.

## 1. Introduction

In recent years, trends in food consumption have experienced changes resulting from factors such as new working patterns, income and the available time that people have. Many studies reveal that current consumer trends, especially in urban areas—the preference for food products that require little preparation, the increase in the purchase of ready cooked meals and more frequent use of home-delivered food—are pushing aside the consumption of more traditional food products [1,2], which have an important role in the traditions of different cultures and regions [3,4].

Traditional food can be defined as products that originate in the agricultural systems that are inherent of a region [5], where local food varieties are habitually used and tend to require a somewhat longer cooking process, but which are incompatible with the current pace of life of many consumers. Not only do such food products contribute to the preservation of traditions, culture and identity of a region [6,7], but they also contribute to rural development and sustainability, as well as to the preservation of biodiversity through the preservation of plant and animal autochthonous varieties, thus supporting rural diversity and social cohesion [8].

Among the most habitual elements of traditional cuisine are spices, which are widely used to add flavour and colour to dishes. However, prior studies analysing whether new consumer trends have affected their use are scarce [9]. The use of spices varies notably from country to country and from region to region, with the most commonly used in Spain being paprika, oregano, cumin, pepper, thyme, rosemary and basil.

Paprika, the traditional spice that is the focus of this study, is one of the most frequently used spices in Spanish kitchens. Its raw material, pepper, originated in America and was brought to Spain by Christopher Columbus in the 15th century [10,11], with paprika production becoming popular in the 16th century through its use as a substitute for the expensive black pepper (*Piper nigrum* L.). Paprika is a widely commercialised spice that is used as flavouring in foodstuffs and as an ingredient in the food industry. Its high colouring power, deriving from the fruit concentration of carotenoids, is an essential aspect which grants it commercial value.

As raw material, the cultivar, ripening stage and growing conditions of the red peppers have an impact on the final carotenoid profile of the paprika [12]. Colour stability and flavour are other quality characteristics of paprika that are determined by the specific processing system and suitable storage conditions [13].

Spain is the third most important producer of red pepper for paprika in the European Union (EU-27), following Romania and Hungary, producing over 6600 t with a value of EUR 20.6 million in the period between 2016 and 2020 [14]. Almost 70% of the total areas and over 60% of the Spanish production comes from *La Vera* region (Southwest Spain). Its produce is known in the markets as *Pimentón de La Vera* smoked paprika.

*La Vera* smoked paprika is a traditional Spanish product whose quality and characteristics, associated with its geographical environment, are preserved by the *Pimentón de La Vera* Protected Designation of Origin (PDO) [15]. The *Pimentón de La Vera* PDO covers a total area of 57,567 ha that extend over 50 municipalities in the natural regions of *La Vera, Campo Arañuelo*, *Valle del Ambroz* and *Valle del Alagón*, in the north of Extremadura, a region in Southwest Spain. The abundance of water, the fertility of the soil and the microclimate of these regions create the ideal conditions to grow peppers to produce paprika.

*Pimentón de La Vera* smoked paprika is obtained by drying the autochthonous pepper varieties (*Capsicum annuum* L.) authorised by the *Pimentón de La Vera PDO* [16]. The thin pericarp of these fruits makes them appropriate for the traditional and laborious drying process based on the burning of oak or holm oak wood as a heat source. Therefore, autochthonous varieties, slow drying and smoking are the differentiating elements of *La Vera* paprika with respect to other types of paprikas, with these processes having significant impact on the sensorial quality and technological properties of this product [17]. The smoke compounds contribute to the aroma and the increase in the antioxidant activity of the paprika [18,19]. As an ingredient, smoked paprika helps stabilise the colour and preserve traditional cured meat products from lipid oxidation throughout the processing stages and storage [20]. Both the area and the production of red pepper local varieties for paprika have increased in Extremadura by more than 35% from 2010 to 2020, with a sustained trend in recent years that confirms the potential of the traditional paprika industry in Spain [21].

As a Protected Designation of Origin, all the production processes for the making of *Pimentón de La Vera* smoked paprika are performed in the farms, drying facilities, mills and packaging facilities situated in this geographical area [22]. Thus, the *Pimentón de La Vera* smoked paprika is recognised for the uniqueness and quality traits associated with the production systems employed, the soil and climatic conditions that are characteristic of the northern valleys of Extremadura, and for processing systems that are unique in the world. This strong link with the territory further strengthens the guarantee of origin, thus ensuring the traceability and quality of the final product. In addition to this, the concentration of agricultural and industrial activities, with the employment of large numbers of workers mainly during the pepper harvesting stage, contributes to the development and sustainability of the rural environment, retaining the population in these less favoured areas, far from the cities.

With this context in mind, the main purpose of this research was to determine whether the new food consumption trends and habits have influenced the consumption of traditional food products such as *Pimentón de La Vera* smoked paprika. In order to do so, we have analysed the consumption of paprika and smoked paprika per inhabitant per year in Spain, taking into account the use of this spice in dishes cooked at home and in restaurants, as well as in the production of dry-cured meat products. Arguably, this study may be able to compensate for the lack of scientific studies on the consumption of this traditional food product, but it may also be useful in the development of rural development policies where maintaining and promoting the making of traditional food products can become a tool to ensure a sustainable economic activity in rural areas. This is particularly applicable in the case of *Pimentón de La Vera* smoked paprika, which has an outstanding role in the economic and social development of northern Extremadura.

## 2. Materials and Methods

### 2.1. Data Collection

Data were collected in November 2022–February 2023 by means of personal interviews with a representative sample of Spanish consumers. The survey was administered by a professional market research company that was responsible for recruiting and training the interviewers and contacting the respondents. The respondents were randomly selected and approached in public areas, such as town squares or shopping centres, where they were informed of the purpose of the study and invited to participate. The whole interview lasted between 8 and 12 min. Quota sampling according to the Spanish demographic criteria of the National Statistics Institute [23] was used regarding age and sex.

The research was conducted in compliance with the University of Extremadura Bio-ethics and Biosecurity Committee regulations regarding studies with human participants. All participants were required to consent to participate in the study and were assured that their responses would be kept confidential and completely anonymous. The respondents did not receive any compensation for their participation in the study.

Considering the topic of research and the differences that could be expected in the purchasing and consumption patterns between consumers from small towns and those from large cities, the surveys were distributed between small towns (less than 55,000 inhabitants), medium-sized towns (55,000–500,000 inhabitants) and large cities (more than 500,000 inhabitants). Following these guidelines, the different locations were selected by the market research company, with the surveys being distributed evenly in order to reach a significant sample in each type of municipality.

Consumers were randomly selected in each of the towns, with surveyors being instructed to interview those responsible for the grocery shopping or cooking in their households, as they were assumed to be more aware of the food being purchased and consumed at home. Prior to the launch of the questionnaire, a pre-test was carried out with 12 participants (not included in the final sample) in order to ensure that the final version of the questionnaire was adequate, clear and neutral [24]. Finally, 1111 completed interviews were obtained, with a maximum range of error of 3.00% for a 95% confidence level (k = 2) which was considered acceptable for these types of studies [25].

The final sample mainly consisted of women (51.93%), people over 50 years of age (49.77%), and people between 36 and 50 years of age (26.46%). These figures were representative of the demographic profile of the Spanish population [23].

### 2.2. Drafting the Questionnaire

Given that paprika consumption occurs mainly in two different spheres (through food recipes that are cooked with paprika and in dry-cured pork sausages whose formulation includes this spice), the questionnaire was designed to obtain information on the consumption of these types of food.

As an introduction, the following information was initially provided to the interviewees:

*Paprika is a spice obtained from red peppers that are dried and ground. It is used both in cooking and as a seasoning ingredient in many food products. Some examples of dishes containing paprika are: ratatouille, Rioja-style potatoes, migas (breadcrumb-based dish), Galician-style boiled octopus, cod, lentil stew, squid and potato stew, bean stew, hummus, beans with pork …, and in dry-cured pork sausages such as chorizo and pork loin.*

*The main types of paprika are smoked (smoke-dried in the traditional style using burning oak or holm oak wood) and unsmoked (sundried or dried using industrial dryers).*

The dishes used as examples were changed according to the geographical area where the interviews were carried out in order to provide the examples that were most familiar to consumers.

Subsequently, respondents were invited to indicate the paprika-based dishes/food products they consumed (excluding sausages) and how often (times/month) they usually consumed them. Another question attempted to obtain specific information on the consumption of dry-cured pork sausages containing paprika, in which this spice is one of the specific ingredients. It should be noted that, within the European Union, Spain is the fifth largest producer of sausages and one of the countries with the highest consumption of these products per inhabitant [26].

Pictures were presented in order to obtain the information on the consumption of paprika-based dry-cured pork sausages. The pictures showed the main types of these sausages together with their average retail weight, and respondents were invited to indicate their monthly consumption. Please see examples of the graphic information provided in Table 1.

Finally, it was also necessary to obtain data on the use of paprika in both cooked food and dry-cured pork sausages. To this end, respondents who cooked at home were asked to indicate which dishes they cooked with paprika, how often they cooked them, for how many people, and how much paprika they used (it was considered that it would be easier for them to indicate the quantities of paprika used in the preparation of each meal rather than per portion). This resulted in a list of dishes being cooked with paprika for which it was possible to determine the average quantity of the spice used in their preparation. With regard to sausages, data from a previous research were used [9]. In that paper, the formulation of different types of traditional dry-cured pork sausages containing paprika (various types of “chorizo” and other dry-cured meats such as pork loin) was determined.

Additionally, the questionnaire also included some questions relating to spice consumption, and PDO paprika use and perceptions, together with a number of socio-demographic questions such as age, sex, level of education or family income.

**Figure 1:**
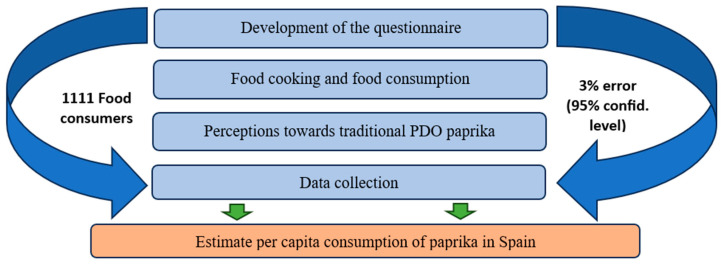
Methodological process.

To date, this is the most comprehensive study on paprika consumption—either smoked or unsmoked—that has been carried out, since this product, due to its consumption levels, is usually included with other flavourings or spices. The study was carried out on the basis of one year’s consumption to avoid seasonal fluctuations, and the data presented correspond to the average of the surveys. Figure 1 summarises the methodological process followed in this research.

## 3. Results and Discussion

The main purpose of this study was to analyse the consumption of paprika in Spain, both common paprika and more specifically smoked paprika. The findings report that the average consumption of paprika per person in Spain is 154.53 per person^−1^ year^−1^. In the case of smoked paprika, the total consumption amounts to 122.16 gr person^−1^ year^−1^. The magnitude of the figures obtained gives an idea of the complexity of this type of study, where the amounts consumed per person are comparatively much smaller than those of other commonly consumed foods. The figures obtained here are lower in comparison to previous studies [9], which was to be expected, since the study carried out by [9] was conducted at a regional level and in the main paprika-producing area of Spain, with consumption expected to be higher there than in other regions of the country.

The total consumption of paprika is broken down into the paprika that is directly consumed in cooked food (62.9%), and that consumed indirectly due to the intake of dry-cured pork sausages that use paprika as a flavouring and preservative (37.1%). Given the scarcity of studies on the consumption of paprika in Spain, the only references for comparison purposes derive from the report on food consumption in Spain of the Ministry of Agriculture, Fisheries and Food [27], which only provides aggregate figures for the consumption of spices and flavourings (150 gr/person and year). This report, however, only takes into account the food purchased by consumers, which means that we can only expect to find lower figures than those obtained in this research. Variations in consumption have been analysed based on the sociodemographic characteristics of the respondents, finding significant differences for some of them, which are shown in Table 2.

The findings reveal that the smaller the town size, the higher the paprika consumption, be it common or smoked paprika. In terms of the age and income variables, the younger the consumer or the lower the income level, the lower the consumption of both types of paprika. However, the level of consumption also decreases among respondents over 65 years of age or among those whose incomes exceed EUR 3000/month. Finally, there are highly significant statistical differences between the consumption of paprika, whether smoked or not, and the size of the families, given that the smaller the size of the family unit, the greater the consumption of this spice.

These results are in line with other research studies that have analysed consumption and preference towards traditional food products. For instance, the study carried out by Cheng et al. [28] revealed that the consumption of traditional food products was higher in small towns, while in larger towns consumers had less time available which led them to consume more convenience food products and, therefore, less home-cooked food. Various studies have also found a lower purchasing frequency of traditional food products among young consumers [29] and less preference for a traditional food product such as honey [30] among younger and lower-income consumers. Subsequently, the respondents were asked to write the paprika brands and designations of origin that they were aware of. The results obtained are shown in Table 3.

The findings reveal that the *Pimentón de La Vera* PDO had the highest percentage of mentions. On the other hand, only 2.13% indicated they were aware of the *Pimentón de Murcia* PDO.

With regards to the brand names mentioned, both producer and own-brand names, 46.05% of the mentions refer to the *Pimentón de La Vera* PDO, which gives an idea of its penetration in the Spanish market. Within all mentions, the second most mentioned category was the distributor’s brand, followed by PDO producer brands. We must highlight that, even in the case of a traditional food product such as paprika, the importance of stores’ own brands was nearly the same as that of producers’ brands, all within a context where consumers demand quality products at better prices and they seem to feel comfortable with stores’ own-brand products [31,32].

Additionally, the answers that numerous participants provided to this question included aspects relating to the attributes of paprika and the geographic region of production. The most frequently commented aspects were sweet and spicy, which are associated with the main types of paprika in the market [33]. Nevertheless, the attribute “smoked”, which is the main differentiating aspect of *La Vera* PDO smoked paprika [20], was very rarely mentioned by consumers, which is a symptom of their lack of familiarity with the unique features of this product. On the other hand, the most commented geographic region was Extremadura, which is in line with the high level of awareness of the *Pimentón de La Vera* PDO that was mentioned earlier on.

Following this, a deeper analysis was performed into the level of awareness of the brand names and PDO for paprika according to the sociodemographic characteristics of the sample. Table 4 shows the findings for sociodemographic characteristics, among which statistically significant differences have been found using *Chi*^2^.

The findings in Table 4 reveal a higher number of mentions of *Pimentón de La Vera* PDO paprika in the largest towns, in comparison to the *Murcia PDO*, which is mentioned in medium-sized and small towns. This might be associated with the leading position of *Pimentón de La Vera* in the Spanish market, which has translated into its significant presence in large distribution chains, habitually found in medium-sized and large towns. Consumers in large towns, unlike those in the smaller towns, tend to use large retailer chains due to their lack of time and their demand for convenience food to suit their working patterns [28].

With respect to brands, respondents between 36–50 years old are the ones who have a greater knowledge of producer brands without PDO and stores’ own brands, while consumers between 51–65 years old have the highest knowledge of PDO brands. The research study conducted by [32] also found that the distributor’s brand is more highly valued by people with lower incomes and lower education levels, as the information on stores’ own brand awareness shows in the previous table.

Finally, participants were given a number of statements regarding PDO paprika with the purpose of analysing the perceptions and attitudes of the Spanish consumers towards this food product. The respondents were invited to state their level of agreement or disagreement with each statement on a scale from 1 (Completely in disagreement) to 5 (Completely in agreement). Table 5 shows the results obtained from the scoring of the various statements broken down by town size.

Generally speaking, consumers from large towns appear to give the worst score to this spice. This fact could be associated to their lower paprika consumption as shown in Table 3. Nevertheless, it is precisely these consumers who give a higher score to the health benefits of this spice. In large towns, the statement “It is a typical ingredient in traditional Spanish cuisine” is given little value, which reflects the new eating habits where convenience food is prioritised [34]. A study carried out by the Ministry of Agriculture, Fisheries and Food [27], also reveals this trend, indicating that in large towns people consume more fast food, whereas in smaller towns, the consumption of food is more centred around the home and traditional food products.

With regards to the attributes “It is a colouring ingredient that is easily replaced with other products” and “I do not trust its authenticity because it is easy to adulterate”, scores over the total are low, which means the respondents do not agree with these statements and thus value PDO paprika’s uniqueness and distinctive role in Spanish cuisine.

Following, Table 6 shows the results of the scores by respondent age.

Taking as a reference the scores shown in Table 5, we can see that young respondents’ (18–35 years old) perceptions towards this spice are the most negative, given that they are the ones who most often think it is expensive, out of date or easily replaced with other products. In addition, in terms of the specific traits of this spice, such as “It is a typical ingredient in traditional Spanish cuisine”, “The PDO is a guarantee of its quality”, “It adds a special colour, aroma and flavour” or “It has effects that are beneficial for health”, the younger consumers gave a lower score to these attributes. These aspects are in contrast with the findings of other studies on food preferences, where no differences were found amongst age groups [35] or where, in fact, the younger respondents gave more importance to the quality label of the food product than other age groups [36]. The difference, however, may be due to the characteristic “easy to replace”, which younger respondents attribute to paprika and can be understood from their perceptions and attitudes.

The age groups between 36 and 65 years of age, which can be considered as the most stable population groups in terms of employment, and therefore, with the higher incomes, are the ones that, to a lesser extent, think that paprika is an expensive spice. On the other hand, consumers over 65 years of age are a potentially inactive population segment, which would lead to a decrease in purchasing power and therefore justify the more negative score of the price factor [37].

Finally, Table 7 shows the results of the scores according to respondent income levels (only those statements where significant differences were found are displayed).

In terms of the scoring of the various statements according to the income level of the respondents, we can see that people with incomes below EUR 1000 are more prone to score the proposed negative aspects highly. On the other hand, respondents with incomes above EUR 2000 tend to perceive this spice more positively.

Interestingly, no significant differences have been found in terms of the consideration of paprika as an expensive spice amongst the different income levels. On a different tone, the score is lower for paprika (out of date, easy to replace) and PDO (I do not trust that it is authentic, PDO as a guarantee of quality) among the lowest income levels, whereas consumers with higher income levels are more appreciative of its typical and traditional nature and trust the authenticity of the product with PDO certification. This result may be associated with the special nature of the product under study, as it is generally assumed that consumers with higher income levels are less price sensitive and give more importance to other attributes relating to quality, such as PDO certification. In fact, research studies such as that of [38] have found that consumers with medium-to-high income levels were more prone to choosing high-quality food products such as PDO-certified products, with these results being similar to those obtained by [39].

## 4. Conclusions

Paprika is a spice that has been traditionally used to flavour foods included in the Mediterranean diet and is closely related to the culture and traditions of several countries such as Spain. Furthermore, pepper production for the purposes of making paprika, as well as the transformation of peppers (initially by the farmer and by the industry at the last stage) is a major economic activity in the rural areas that produce it, thus contributing to sustainable rural development.

However, the new trends in food consumption, where consumers demand more convenience food in order to make best use of the little time they have for cooking, pose barriers to the consumption of this spice—as is the case with other traditional food products—and preventing it from staying in the diet, especially among the new generations.

In this sense, this research is innovative, as it analyses Spanish consumers’ perceptions towards paprika as a traditional food product, as well as their per-capita consumption of this spice. These aspects have been scarcely studied given the low per-capita consumption of these type of products. In fact, the average consumption of paprika found in this study is only 154.53 g per person per year, and for the most traditional smoked paprika, only 122.16 g per person per year. These figures are in line with other studies and reveal the difficulty of studying the consumption of some foodstuffs which, although strongly linked to culinary traditions, have substantial differences in their consumption levels compared to other more common foods in the diet.

Generally speaking, the perception of paprika is better in small towns and amongst older consumers, which has translated into a higher level of consumption, reflecting a trend to consume more traditional food products in these particular segments. This also unveils the issues to attract new consumers (currently young consumers who live in larger towns and buy from modern distribution chains) who might be able to maintain the production and consumption of the product in the future.

With regards to the role of the PDO label in the consumption of paprika, the *Pimentón de La Vera* PDO is seen to be the leader in Spain for this product amongst all the consumer types, and especially consumers living in large and medium-sized towns. However, a lack of awareness is perceived on the special characteristics of this “smoked” paprika.

These results confirm the change in food consumption patterns, where products made using traditional methods are losing presence and competitiveness amongst modern consumers. This fact could be the basis for future research relating to the maintenance and sustainability of rural areas, as well as for the design of marketing strategies and policies which could help traditional agri-food companies.

Based on the importance of traditional foods at economic and social levels, and especially in rural areas, it is seen as necessary to promote these types of food products, such as paprika, since they contribute to ensuring sustainability and livelihoods. Although the consumption of these products has been seen to decrease, it should be noted that they evoke a sense of belonging, both to the local community and the family. This can become an excellent basis for a marketing strategy that could provide additional information about the culture and traditions relating to the product, and its positive effects for the care and strengthening of the community.

### Theoretical and Practical Implications

This work has theoretical and practical implications. Regarding the former, it contributes to the literature on the consumption of traditional foods in Spain. Although studies on changes and trends in the consumption of traditional foods in Spain already exist, they are scarce, especially in relation to spices and condiments [1,2,3,4,5].

In terms of its practical implications, they can be divided between those aimed at the public sector and those in the private sector. On the public sector side, results will inform governments and public administrations on changing trends in the consumption of traditional foods to enable the development of guidelines to address these changes in a sustainable and balanced way, adapted both to the specific needs of the food sector and to those the general public.

In addition, the suggestions for the public sector that are extracted from this research could also be developed through promotional campaigns aimed at raising awareness, and sensitisation to and knowledge of traditional food products that seek to promote new eating patterns. Another implication of this study may be the development of policies that promote rural and sustainable development and the potential improvement of the quality of life of local communities. Adequate public policies would be effective in developing and strengthening the resilience and adaptability of local communities as a pathway to sustainable development.

Lastly, the results of this study also have implications for the agri-food sector, specially concerning the development of non-technological and market-oriented innovation strategies to ensure the sustainability of their production in the face of fluctuations and changing trends in food consumption.

## Figures and Tables

**Table 1:** Please indicate your average consumption of the following types of sausages.

	Sample	Consumption (Grams/Month)
Red chorizo in gut casing Approximate retail weight 900 gr	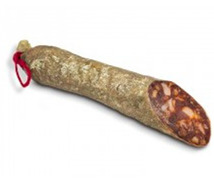	
Horseshoe red chorizo Approximate retail weight 350 gr	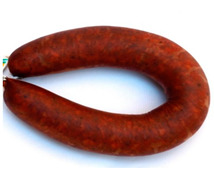	
Smoked red chorizo Approximate retail weight 500 gr	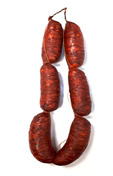	

**Table 2:** Consumption of paprika and smoked paprika based on sociodemographic and habitat characteristics (gr/person per year).

		Paprika Consumption (gr Person^−1^ Year^−1^)	Smoked Paprika Consumption (gr Person^−1^ Year^−1^)
Town size (**/***)	<55,000 inhabitants	167.24	137.13
	55,000–500,000 inhabitants	151.93	134.55
	>500,000 inhabitants	148.19	97.79
Age (***/***)	18–35 years	139.27	104.41
	36–50 years	163.03	130.16
	51–65 years	166.12	135.82
	>65 years	148.03	116.50
Family size (***/***)	1–2 people	164.17	129.50
	3–4 people	147.78	119.40
	≥persons	121.57	80.97
Income (**/*)	<EUR 1000/month	145.06	110.26
	EUR 1001–2000/month	155.05	126.12
	EUR 2001–3000/month	178.58	139.97
	>EUR 3000/month	147.25	125.38

Significance at: * *p* < 0.1, ** *p* < 0.05, *** *p* < 0.001for both paprika/smoked paprika consumption.

**Table 3:** Awareness of paprika brands and PDO (% of mentions).

Category	Subcategory	%
Designation of Origin	La Vera	46.05
	Murcia	2.23
Producer Brand Names	PDO	16.25
	No PDO	3.82
Store own brand/Distributor		17.22
Attributes	Sweet	0.90
	Spicy	0.75
	Others (smoked, organic, savoury, …)	0.76
Geographic Area	Extremadura	2.29
	Others (La Rioja, Italian, …)	0.70
Unaware		9.03

**Table 4:** Awareness of brand names and PDO according to sociodemographic aspects (%).

		Town Size ***			Age ***				Education Level ***				Income (EUR)***			
Category	Subcategory	Large	Medium	Small	18–35	36–50	51–65	>65	Primary	Secondary	Upper Secondary	University	<1000	1000–2000	2000–3000	>3000
Designation of Origin	La Vera	25.34	44.49	30.17	16.59	30.62	29.11	23.68	15.54	11.46	29.41	43.59	19.38	41.10	24.21	15.31
	Murcia	12.50	43.75	43.75	12.50	18.75	43.75	25.00	10.94	7.81	45.31	35.94	18.75	28.13	40.62	12.50
Producer Brand Names	PDO	51.28	35.04	13.68	12.39	28.63	32.05	26.93	26.07	19.66	21.79	32.48	11.52	61.21	21.82	5.45
	No PDO	27.27	58.18	14.55	10.91	36.36	23.64	29.09	27.28	7.27	27.27	38.18	8.82	61.76	26.47	2.95
Store own brand/ Distributor		66.94	19.35	13.71	24.60	31.05	22.58	21.77	17.34	17.74	32.66	32.26	11.98	53.13	28.12	6.77
Attributes	Sweet	23.08	69.23	7.69	0.00	7.69	38.46	53.85	11.54	26.92	42.31	19.23	25.00	55.77	9.62	9.61
	Spicy	27.27	72.73	0.00	0.00	9.09	45.45	45.45	13.64	31.82	40.91	13.63	31.82	40.91	13.64	13.63
Geographic Area	Extremadura	44.44	48.15	7.41	29.63	11.11	7.41	51.85	21.30	50.93	17.59	10.18	21.30	50.93	17.59	10.18
Unaware		16.15	47.69	36.16	59.23	10.00	12.31	18.46	13.27	17.12	52.50	17.11	38.46	23.08	20.00	18.46

Significance at: *** *p* < 0.001.

**Table 5:** Scoring for the attributes of PDO paprika according to town size (scale from 1: Completely in disagreement to 5: Completely in agreement).

	Town Size			
	Small	Medium	Large	Total
It is a typical ingredient in traditional Spanish cuisine ***	4.31	4.33	4.06	4.23
It is an expensive spice ***	2.12	2.25	2.05	2.15
It is a colouring ingredient that is easily replaced with other products	2.42	2.32	2.26	2.33
It is an out-of-date spice ***	1.90	1.99	2.11	2.01
I do not trust its authenticity because it is easy to adulterate	2.78	2.81	2.81	2.80
The PDO is a guarantee of its quality ***	4.06	4.28	3.91	4.09
It is a gourmet-only ingredient or for cooking experts **	1.84	1.73	1.85	1.80
It adds a special colour, aroma and flavour ***	4.31	4.52	4.37	4.42
It has effects that are beneficial for health **	3.40	3.42	3.53	3.46

Significance at: ** *p* < 0.05, *** *p* < 0.001; n.s.: non-significant.

**Table 6:** Scoring for the attributes of PDO paprika by age (scale from 1: Completely in disagreement to 5: Completely in agreement).

	18–35 Years	36–50 Years	51–65 Years	>65 Years
It is a typical ingredient in traditional Spanish cuisine (***)	4.00	4.29	4.30	4.32
It is an expensive spice (***)	2.31	2.09	2.03	2.18
It is a colouring ingredient that is easily replaced with other products (***)	2.67	2.23	2.17	2.25
It is an out-of-date spice (***)	2.32	1.89	1.92	1.92
I do not trust its authenticity because it is easy to adulterate (***)	2.98	2.71	2.77	2.75
The PDO is a guaranty of its quality (***)	3.86	4.17	4.16	4.16
It is a gourmet-only ingredient or for cooking experts (***)	1.89	1.69	1.76	1.88
It adds a special colour, aroma and flavour (***)	4.29	4.51	4.43	4.43
It has effects that are beneficial for health (***)	3.31	3.52	3.54	3.44

Significance at: *** *p* < 0.001.

**Table 7:** Scoring for the attributes of PDO paprika by income level (scale from 1: Completely in disagreement to 5: Completely in agreement).

	<1000 EUR/Month	1001–2000 EUR/Month	2001–3000 EUR/Month	>3000 EUR/Month
It is a typical ingredient in traditional Spanish cuisine (***)	4.23	4.08	4.35	4.44
It is a colouring ingredient that is easily replaced with other products (***)	2.55	2.18	2.24	2.37
It is an out-of-date spice (***)	2.29	2.01	1.82	1.75
I do not trust its authenticity because it is easy to adulterate (**)	2.92	2.83	2.68	2.57
The PDO is a guarantee of its quality (***)	3.86	4.08	4.31	4.13

Significance at: ** *p* < 0.05, *** *p* < 0.001.

## Data Availability

The data presented in this study are available on request from the corresponding author.
